# Can misfit dislocations be located above the interface of InAs/GaAs (001) epitaxial quantum dots?

**DOI:** 10.1186/1556-276X-7-486

**Published:** 2012-08-31

**Authors:** Zi-Bin Chen, Wen Lei, Bin Chen, Yan-Bo Wang, Xiao-Zhou Liao, Hoe H Tan, Jin Zou, Simon P Ringer, Chennupati Jagadish

**Affiliations:** 1 School of Aerospace, Mechanical and Mechatronic Engineering, The University of Sydney, Sydney, New South Wales, 2006, Australia; 2 School of Electrical, Electronic and Computer Engineering, The University of Western Australia, Perth, Western Australia, 6009, Australia; 3 Department of Electronic Materials Engineering, Research School of Physics and Engineering, The Australian National University, Canberra, Australian Capital Territory, 0200, Australia; 4 Materials Engineering and Centre for Microscopy and Microanalysis, The University of Queensland, Brisbane, Queensland, 4072, Australia; 5 Australian Centre for Microscopy and Microanalysis, The University of Sydney, Sydney, New South Wales, 2006, Australia

**Keywords:** Quantum dot, Electron microscopy, Misfit dislocations, Droplet epitaxy, Oxidation

## Abstract

InAs/GaAs(001) quantum dots grown by droplet epitaxy were investigated using electron microscopy. Misfit dislocations in relaxed InAs/GaAs(001) islands were found to be located approximately 2 nm above the crystalline sample surface, which provides an impression that the misfit dislocations did not form at the island/substrate interface. However, detailed microscopy data analysis indicates that the observation is in fact an artefact caused by the surface oxidation of the material that resulted in substrate surface moving down about 2 nm. As such, caution is needed in explaining the observed interfacial structure.

## Background

Semiconductor quantum dots (QDs) are attracting increasing research interest because of their important potential applications in electronic and optoelectronic devices [1-4]. Among the many techniques to produce QDs, coherent island formation through heteroepitaxial growth of semiconductor materials has been the most important technique because of the possible combination of the QD growth and semiconductor integration techniques. Two different methods have been used to grow epitaxial QDs. The first one is the classical Stranski-Krastanow (S-K) growth, [5,6] in which a semiconductor material with larger lattice parameters is first deposited on the substrate with smaller lattice parameters layer-by-layer, forming a wetting layer, followed by island formation to partially release the strain energy caused by the lattice mismatch between the epilayer and the substrate. The other method is droplet epitaxy [7-9] that has been used for the growth of III-V semiconductor QDs [10,11] by firstly introducing liquid nanodroplets of the group III element on the substrate and then exposing the droplets to a gas-phase flow of the group V element. Different from the S-K growth mode, droplet epitaxy does not depend on lattice mismatch and therefore can be applied to more materials systems.

For both the S-K growth and the droplet epitaxy, misfit dislocations will be introduced to further release the strain caused by the lattice mismatch when the size of a QD reaches a critical value [12,13]. Because misfit dislocations produce deleterious effects on QD properties, the mechanisms on how misfit dislocations are generated have been extensively investigated both experimentally and theoretically [11-20]. Based on the fact that the highest stress in a QD occurs at the corner where the island meets the substrate, it has been suggested that strain-relieving perfect misfit dislocations are generated at the island edge, when the island reaches its critical size [14-16]. The generation and morphology of perfect misfit dislocations have been considered theoretically [17-19]. Partial misfit dislocations have also been identified in relaxed islands [9,20] and are believed to be energetically more favourable than perfect misfit dislocations in some regions of QDs [21]. In some situations, e.g. Ge/Si(001) QDs grown at high temperature, partial misfit dislocations can originate from the surface of QD islands and then glide to the island/substrate interface [22]. While most misfit dislocations are located at the island/substrate interface, it is interesting to note an exception in a recent report that presented evidence of misfit dislocations located above the island/substrate interface in an S-K-grown GaSb/GaAs(001) system [23]. The phenomenon was explained based on the compressive stress induced on the GaSb islands by the GaAs substrate due to lattice mismatch between the epilayer and the substrate [23]. In this letter, we conducted electron microscopy characterization of InAs/GaAs(001) QDs grown by droplet epitaxy. Misfit dislocations in relaxed InAs/GaAs(001) islands were found to be located about 2 nm above the island/substrate interface. However, detailed analysis of the transmission electron microscopy (TEM) data indicates that the observation is in fact an artefact caused by surface oxidation of the material that resulted in substrate surface moving down about 2 nm. We therefore conclude that caution is needed when explaining the interfacial structure of the QDs.

## Methods

A double-layer InAs/GaAs(001) QD sample was grown by droplet epitaxy using metal organic vapour phase epitaxy. The QD sample was grown on a semi-insulating GaAs(001) substrate in a horizontal flow reactor (AIX200/4, AIXTRON SE, Herzogenrath, Germany) at a pressure of 100 mbar. Trimethylindium, trimethylgallium and AsH_3_ were used as the precursors and ultrahigh-purity H_2_ as the carrier gas. Firstly, a 200-nm GaAs buffer layer was deposited at 650°C, then the temperature was reduced to 500°C and the growth was interrupted for 10 s with all sources removed from the reactor to eliminate the influence of AsH_3_ source on the later deposition of indium droplets. After that, two monolayers of indium droplets were deposited, which were then immediately exposed to the AsH_3_ flow (3.0 × 10^−4^ mol/min) for 15 s. The InAs QDs were capped immediately by a 10-nm GaAs capping layer at 500°C. Then, the growth temperature was ramped up to 650°C and a 100-nm GaAs layer was deposited. The InAs QDs were capped immediately by a 100-nm GaAs capping layer whilst the temperature was ramped up to 650°C. Finally, a surface layer of InAs QDs was grown in the same fashion as the buried InAs QDs.

Only the structures of QDs on the top layer were investigated. Cross-sectional TEM specimens were prepared using a Gatan precision ion polishing system (Gatan, Inc., Pleasanton, CA, USA) with Ar^+^ energy of 2.5 keV. Structural characterization of the QDs was conducted using scanning electron microscopy (SEM; Zeiss Ultra+, Carl Zeiss, Inc., Oberkochen, Germany) operated at 2 kV and TEM (JEM-3000 F, JEOL Ltd., Akishima-shi, Japan) operated at 300 kV. Quantitative compositional analysis was conducted using X-ray energy-dispersive spectroscopy (XEDS) in JEM-2200 TEM (JEOL Ltd.) operated at 200 kV and the ESPRIT software. The electron probe size used for the XEDS was 1 nm. XEDS data collection time was controlled to make sure that high counts (larger than 10 thousands) were obtained for datum points so that the statistical errors were less than 1%.

## Results and discussion

**Figure 1:**
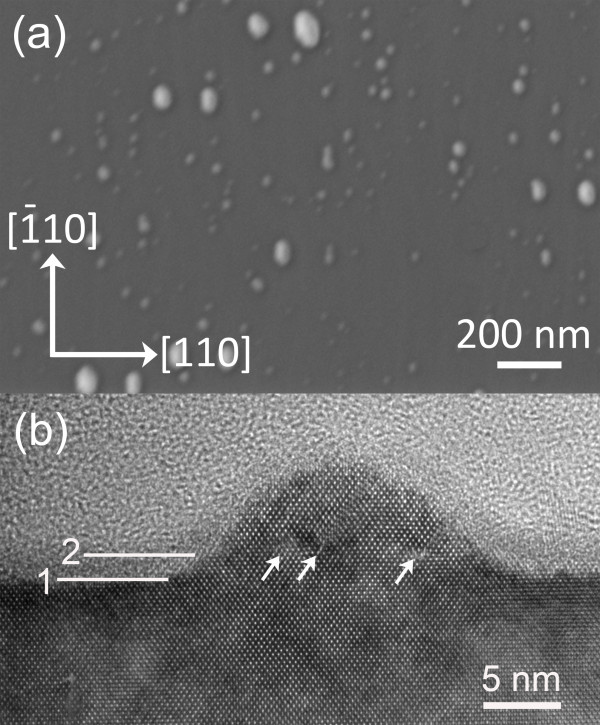
**Morphology of the QDs.** (**a**) An SEM image of the top surface of the InAs/GaAs(001) QDs; (**b**) a [$\bar{1}10$] cross-sectional TEM image showing a relaxed InAs island on the GaAs(001) substrate. Line 1 and line 2 indicate the positions of the crystalline surface of the substrate and the surface of the dark amorphous layer, respectively. Three misfit dislocations in the relaxed island are marked with three white arrows.

Figure 1a shows a typical plan-view SEM image of the QDs on the surface. Large islands with widths (measured along the [110] direction) larger than approximately 30 nm are usually elongated and faceted, showing typical strain-relaxed morphologies [24]. Most of small islands with widths smaller than approximately 15 nm have an elliptical shape, although a small number of them are circular. The large islands are all relaxed through the formation of misfit dislocations. Figure 1b shows a typical $\overset{―}{1}10$ cross-sectional high-resolution TEM image of a relaxed large island on the sample surface. Three misfit dislocations, which are indicated with three white arrows, are seen at the same atomic layer in the island, which is approximately 2 nm above the crystalline surface of the substrate. A white line marked as ‘1’ indicates the crystalline surface of the substrate, and the other white line marked with ‘2’ specifies the position of the atomic layer on which the misfit dislocations lie.

The phenomenon of misfit dislocations positioned above the crystalline substrate surface seen in Figure 1 is very similar to the phenomenon reported in [23]. However, another interesting phenomenon is also seen in Figure 1. There are two amorphous structures with distinctly different contrasts above the crystalline structure: one is a thin amorphous layer with a thickness of about 2 nm immediately above the crystalline substrate and island surface and with contrast clearly darker than the other part of the amorphous area above the sample. The top surface of the 2-nm thin amorphous layer is located at exactly the same level as the atomic plane where misfit dislocations lie, i.e. the layer indicated by line 2. While the amorphous structure with bright contrast is the epoxy used for cross-sectional sample preparation, it is not clear what the dark amorphous structure is.

**Figure 2:**
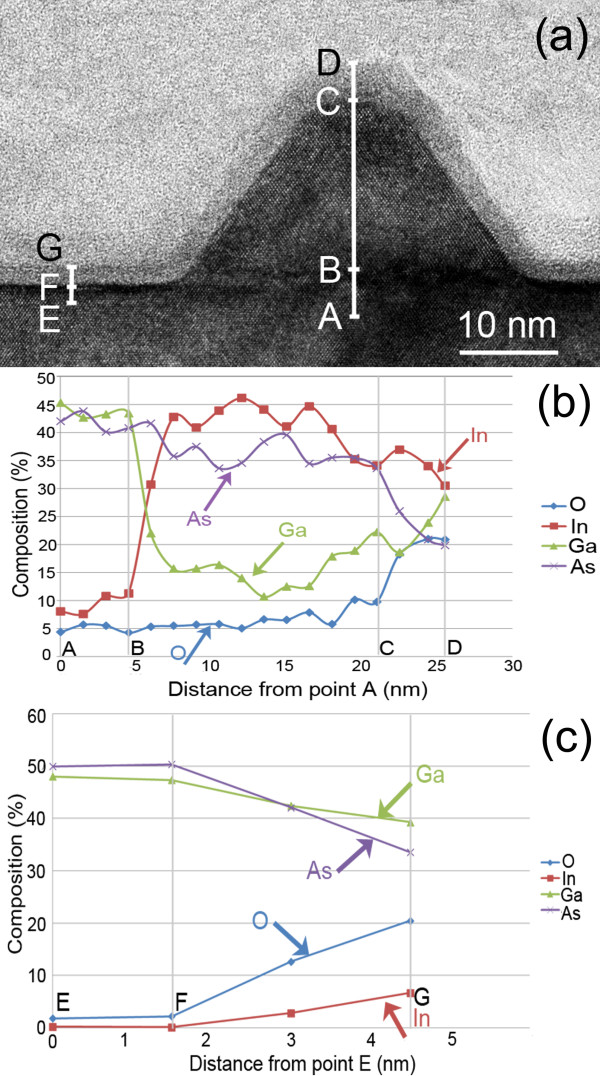
**XEDS analysis.** (**a**) A [$\bar{1}10$] cross-sectional TEM image of a relaxed InAs island. Straight lines ABCD and EFG indicate the positions from which XEDS data were obtained; (**b**) XEDS data detected along the line ABCD; (**c**) XEDS data detected along the line EFG.

XEDS analysis was conducted to explore the composition of the dark-contrast amorphous layer and surrounding areas. Figure 2 shows the results obtained from an island and its nearby area. Line scans were conducted along lines ABCD and EFG shown in Figure 2a with the interval of the datum points at approximately 2 nm. O, Ga, As and In were detected in the material. The quantitative concentrations of these elements as a function of positions along lines ABCD and EFG are shown in Figure 2b,c, respectively. From Figure 2b, a small amount of In content is detected in the substrate; this is caused by the combined effect of the contamination induced during the sample preparation process and the electron beam spreading to the island through the scatter of the electron beam in the TEM sample. The latter effect can be removed when XEDS was conducted in the substrate far away from the island, as shown in Figure 2c. Another possible reason that In is detected in the substrate is the mutual diffusion of In into the substrate and Ga into islands during the high-temperature sample deposition process. This explains the phenomenon that over 10% of Ga is detected in the island. The inter-diffusion process of In and Ga is in fact an effective way to reduce the strain energy of the system [16,25,26].

The most interesting phenomena revealed by the XEDS data in Figure 2 are (1) although O is detected everywhere in the sample, which is caused by surface oxidation after TEM sample preparation, the O content increases significantly at the dark amorphous layer; (2) the contents of Ga at the position immediately below misfit dislocations and at the dark-contrast amorphous layer immediately above the crystalline substrate are very high; and (3) the In content at the dark-contrast amorphous layer covering the island is very high. All these evidence point to the conclusion that the dark-contrast amorphous layer is actually an oxidised layer that was originally the surface of the crystalline GaAs substrate and the surface of InAs islands. The oxidised amorphous layer formed after the epitaxial sample surface was exposed to the air and therefore was only seen on the sample surface, not in the buried QD layer. Therefore, misfit dislocations in large relaxed QD islands formed exactly at the interface of the epilayer and the substrate, not at a level approximately 2 nm above the interface as it looks in Figure 1. Because oxidation of the surface of semiconductor nanostructures has been widely reported in literatures [27-29], not just in the InAs/GaAs QD structure reported here, caution is needed when investigating the interfacial structures of epitaxial materials.

## Conclusions

In summary, misfit dislocations in InAs/GaAs(001) QDs grown by droplet epitaxy are observed to be located approximately 2 nm above the crystalline substrate surface. However, detailed compositional analysis suggests that this is an artefact caused by surface oxidation. The oxidised surface is of an amorphous structure with a thickness of approximately 2 nm.

## Competing interests

The authors declare that they have no competing interests.

## Authors’ contributions

ZBC, WL and XZL designed the study. ZBC conducted the microscopy experiments. WL, HHT and CJ grew the QD specimens. ZBC and XZL wrote the paper. All authors discussed the results and contributed to the paper. All authors read and approved the final manuscript.

## Authors’ information

ZBC is a postgraduate student. Dr WL is a research associate professor. Dr BC is a postdoctoral research associate. Dr YBW is an Australian Research Council (ARC) Australian Postdoctoral Fellow. Dr XZL is an associate professor and ARC Future Fellow. Dr HHT is a senior fellow and ARC Future Fellow. Dr JZ is a professor and ARC Future Fellow. SPR is the Director of the Australian Centre for Microscopy and Microanalysis, the Executive Director and CEO of the Australian Microscopy and Microanalysis Research Facility, and the Director of the Bandwidth Foundry International Pty Ltd. Dr CJ is a distinguished professor and ARC Laureate Fellow.
